# Xeno-free cryopreservation of adherent retinal pigmented epithelium yields viable and functional cells in vitro and in vivo

**DOI:** 10.1038/s41598-021-85631-6

**Published:** 2021-03-18

**Authors:** Britney O. Pennington, Jeffrey K. Bailey, Mohamed A. Faynus, Cassidy Hinman, Mitchell N. Hee, Rory Ritts, Vignesh Nadar, Danhong Zhu, Debbie Mitra, Juan Carlos Martinez-Camarillo, Tai-Chi Lin, Biju B. Thomas, David R. Hinton, Mark S. Humayun, Jane Lebkowski, Lincoln V. Johnson, Dennis O. Clegg

**Affiliations:** 1 Center for Stem Cell Biology and Engineering, Neuroscience Research Institute, University of California, 6131 Biology 2 Bldg 571, NRI, UC Santa Barbara, Santa Barbara, CA 93106 USA; 2 Regenerative Patch Technologies LLC, Portola Valley, CA USA; 3 College of Creative Studies, Biology, University of California, Santa Barbara, CA USA; 4 Department of Molecular Cellular and Developmental Biology, University of California, Santa Barbara, CA USA; 5 Department of Pathology and Ophthalmology, USC Roski Eye Institute, Keck School of Medicine of the University of Southern California, Los Angeles, CA USA; 6 Department of Biomedical Engineering, Denney Research Center (DRB) of the University of Southern California, Los Angeles, CA USA; 7 USC Dr. Allen and Charlotte Ginsburg Institute for Biomedical Therapeutics, University of Southern California, Los Angeles, CA USA

**Keywords:** Regenerative medicine, Stem-cell biotechnology, Tissue engineering

## Abstract

Age-related macular degeneration (AMD) is the primary cause of blindness in adults over 60 years of age, and clinical trials are currently assessing the therapeutic potential of retinal pigmented epithelial (RPE) cell monolayers on implantable scaffolds to treat this disease. However, challenges related to the culture, long-term storage, and long-distance transport of such implants currently limit the widespread use of adherent RPE cells as therapeutics. Here we report a xeno-free protocol to cryopreserve a confluent monolayer of clinical-grade, human embryonic stem cell-derived RPE cells on a parylene scaffold (REPS) that yields viable, polarized, and functional RPE cells post-thaw*.* Thawed cells exhibit ≥ 95% viability, have morphology, pigmentation, and gene expression characteristic of mature RPE cells, and secrete the neuroprotective protein, pigment epithelium-derived factor (PEDF). Stability under liquid nitrogen (LN_2_) storage has been confirmed through one year. REPS were administered immediately post-thaw into the subretinal space of a mammalian model, the Royal College of Surgeons (RCS)/nude rat. Implanted REPS were assessed at 30, 60, and 90 days post-implantation, and thawed cells demonstrate survival as an intact monolayer on the parylene scaffold. Furthermore, immunoreactivity for the maturation marker, RPE65, significantly increased over the post-implantation period in vivo, and cells demonstrated functional attributes similar to non-cryopreserved controls. The capacity to cryopreserve adherent cellular therapeutics permits extended storage and stable transport to surgical sites, enabling broad distribution for the treatment of prevalent diseases such as AMD.

## Introduction

Age-related macular degeneration (AMD) affects over 190 million people worldwide^1^ and is the leading cause of blindness in the elderly population of European descent^2^. Vision loss due to non-neovascular AMD is associated with the degeneration of the retinal pigmented epithelial (RPE) cells and the consequent dysfunction of the overlying photoreceptors in the macula, the region of the retina responsible for high acuity vision^3^. In an effort to ameliorate the effects of non-neovascular AMD and to delay the progression of this disease, for which no approved treatment currently exists, one therapeutic strategy involves delivery of exogenous RPE cells into the subretinal space to replace the degenerated native epithelium^4^. Recent advancements in regenerative medicine have identified human pluripotent stem cells (PSC) as a potentially unlimited source of therapeutic RPE cells^3^. Pre-clinical animal studies have demonstrated that PSC-derived RPE cells may survive and integrate with the host RPE when injected as a cellular suspension^5,6^ and that survival and retinal interaction is improved when RPE cells are implanted as an epithelial monolayer supported by a substrate^7,8^.

Therapeutic implants comprised of PSC-derived RPE cells supported by a proteinaceous sheet or an implantable scaffold also offer the advantage of delivering a monolayer of mature RPE cells specifically to a diseased region in the eye^4,9^. Results from multiple Phase I clinical trials suggest that such implants represent a promising approach for the treatment of degenerative eye diseases^10–14^. In 2018, Kashani et al. reported interim results from a Phase I/IIa clinical trial, which found that vison loss did not progress in AMD patients who received human embryonic stem cell (hESC)-derived RPE cells seeded on a parylene-C scaffold^10^. Parylene-C is an inert, biostable polymer commonly used in biomedical applications such as coating stents, pacemakers, and retinal prostheses^15,16^ and has been engineered to support RPE cells^9,17–19^. Furthermore, post-operative data from the Kashani et al. study suggest that implanted RPE cells can support the host neural retina and improve the fixation capabilities of the implanted eye compared to the untreated, contralateral eye^10^.

RPE implants currently employed in clinical trials are maintained in culture until the time of administration, which severely limits shelf life, restricts transport to treatment sites, and requires the coordination of surgical procedures with product manufacture. Cryopreservation of RPE-seeded implants would significantly increase product availability by extending shelf-life, increasing the flexibility of manufacturing and clinical schedules, and enabling wide-spread on-demand distribution. The capacity to cryopreserve these implants and administer an adherent cellular therapy immediately post-thaw has been identified as a crucial milestone for commercialization of such therapies^4^. However, previous attempts to cryopreserve or vitrify adherent monolayers of mammalian cells have resulted in highly variable (35–89%) post-thaw survival rates^20–23^, and no clinical application of a cryopreserved, adherent RPE cell-based therapy has yet been reported.

Here we describe a novel, xeno-free method for the successful cryopreservation and thaw of an adherent monolayer of hESC-derived Retinal pigmented Epithelial cells on a Parylene Scaffold (REPS) and perform in vitro and in vivo characterization of REPS produced using this protocol. Several cryopreservation parameters were investigated including freeze rate, concentration of cryoprotective agent, and post-thaw rinse solution. Surprisingly, the most important factor for the successful cryopreservation of REPS was the duration of adherent cell culture prior to the time of freeze. Clinical-grade REPS cryopreserved using the optimized parameters reproducibly exhibit ≥ 95% post-thaw survival, robust expression of RPE-specific genes, neurotrophic factor secretion, as well as demonstrating stability through one year of storage in liquid nitrogen (LN_2_). Furthermore, indications of RPE cell survival, maturation, and phagocytic function in vivo were observed for REPS that were immediately implanted into the subretinal space post-thaw. Together, these findings provide a path toward the clinical manufacture and implementation of cryopreserved adherent RPE-based therapeutics.

## Results

### Optimization of cryopreservation parameters for REPS

**Figure 1:**
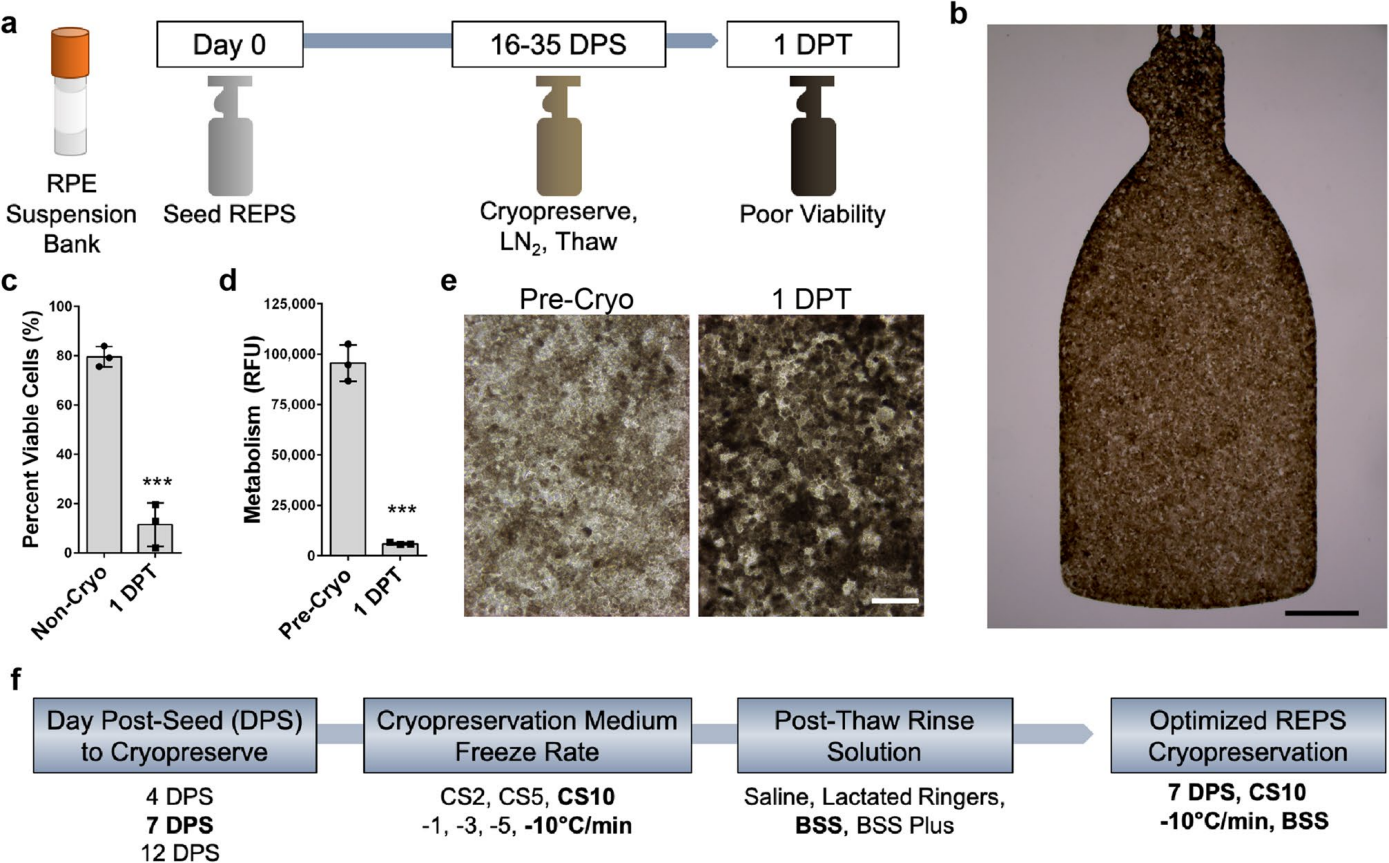
Pigmented REPS exhibit poor cell survival in response to cryopreservation. (**a**) Schematic of overall process to seed, cryopreserve, and thaw a mature monolayer of Retinal pigmented Epithelial cells adhered to a Parylene Scaffold (REPS). (**b**) Representative image of REPS exhibiting pigmentation at 28 days post-seeding (DPS) (Bright field; scale bar, 1 mm). (**c–e**) REPS were cryopreserved at 30 DPS. (**c**) Cryopreservation of pigmented REPS results in significantly reduced viability one day post-thaw (DPT) compared to the non-cryopreserved control. (**d**) Pigmented REPS exhibit significantly reduced metabolic activity 1 DPT compared to levels measured prior to cryopreservation. Relative Fluorescence Units (RFU). (**e**) Cryopreservation of pigmented REPS results in severe hyperpigmentation within the first 24 hours post-thaw (Bright field; scale bar, 100 µm). (**f**) Optimized parameters to cryopreserve REPS were identified by analysis of (i) post-seeding culture period, (ii) cryopreservation medium and freeze rate, and (iii) post-thaw rinse solution. Optimal parameters are identified in bold (see also Supplementary Figs. S1, S2). Error bars indicate standard deviation. ***P < 0.001, unpaired two-tailed t-test.

REPS are produced by seeding a suspension of RPE cells onto a vitronectin-coated parylene scaffold followed by in vitro culture until the time of cryopreservation (Fig. 1a). Like other melanized cell types, RPE cells develop melanosome organelles, which are the intracellular sites of melanin biosynthesis. After approximately two weeks of culture, REPS exhibit cellular pigmentation, and cells gradually appear darker by visual inspection with additional time in culture. We initially generated and attempted to cryopreserve REPS that were similar to the mature, non-cryopreserved RPE implant employed in a Phase I/IIa clinical trial^10^ (Fig. 1a,b). However, attempts to cryopreserve pigmented REPS were unsuccessful (Fig. 1c–e). By one day post-thaw (DPT) less than 10% of the RPE cells were viable as determined by propidium iodide exclusion and exhibited significantly lower metabolic rates compared to pre-freeze levels as assessed by AlamarBlue reduction (*P* < 0.001) (Fig. 1c,d,). Notably, low post-thaw viability was associated with rapid hyperpigmentation that manifested within the first 24 hours post-thaw (Fig. 1e). Due to the poor post-thaw phenotype of pigmented RPE cells, we developed a process to cryopreserve REPS by (i) identifying a time point to freeze REPS prior to the onset of RPE cell pigmentation, (ii) determining an optimal cryopreservation medium and freeze rate, and (iii) selecting a compatible post-thaw rinse solution (Fig. 1f).

To determine the optimal post-seeding culture period, REPS were cryopreserved at 4, 7, and 12 days post-seeding (DPS) and were unpigmented by visual inspection (Supplementary Fig. S1a). Morphology and gene expression were assessed prior to cryopreservation, immediately post-thaw, and following 30 days of culture post-thaw. Since stress associated with the freeze/thaw processes could lead to cell loss and induce epithelial-to-mesenchymal transition (EMT), the effect of cryopreservation on the expression of genes abundant in early (*PMEL*) and mature (*RPE65, RLBP1*) RPE cells, as well as those indicative of EMT (*S100A4*) was assessed. REPS cryopreserved at 7 DPS yielded optimal results as exhibited by similar expression levels of RPE marker genes immediately post-thaw compared to 30 DPT and maintenance of typical RPE cuboidal morphology throughout the course of analysis (Supplementary Fig. S1a,b), which is desirable for a therapeutic intended for implantation immediately post-thaw. Conversely, implants cryopreserved at 4 DPS or 12 DPS expressed either significantly higher *S100A4* (*P* < 0.01) or significantly lower *RPE65* (*P* < 0.05), respectively, when assessed immediately post-thaw compared to 30 DPT (Supplementary Fig. S1b). Both 4 DPS and 12 DPS conditions also displayed fibroblastic cell morphology 1 DPT (Supplementary Fig. S1a). Although all conditions were able to recover a pigmented, cuboidal appearance by 30 DPT with gene expression profiles similar to mature, non-cryopreserved control REPS (Supplementary Fig. S1a,b), downstream optimization employed REPS cryopreserved at 7 DPS to develop a product ready for immediate administration post-thaw.

Appropriate freezing parameters depend on available cell surface area, rate of fluid transport across the plasma membrane, and the permeability of the scaffold^18,24^. These properties, combined with the cooling rate and the concentration of cryoprotective agents such as dimethyl sulfoxide (DMSO), affect intracellular ice formation, osmotic potential, and cell survival post-thaw^24^. REPS were cryopreserved using CryoStor, a fully defined, xeno-free cryopreservation medium. Formulations of CryoStor containing 2%, 5%, and 10% DMSO (CS2, CS5, and CS10, respectively) were tested using freeze rates of − 1 °C/min, − 3 °C/min, − 5 °C/min, and − 10 °C/min. Since the cumulative effects of necrotic and apoptotic pathways in response to freeze/thaw manifest within the first 24 hours after thaw^25^, cell viability was assessed 1 DPT in all cases. Although no significant differences in post-thaw viability were detected among the conditions (*P* = 0.078), qualitative differences in RPE cell morphology were observed by visual inspection (Supplementary Fig. S1c,d). REPS frozen in CryoStor formulations that contained higher concentrations of DMSO (CS5 and CS10) exhibited more consistent cuboidal RPE cell morphology 1 DPT compared to REPS frozen in CS2 (Supplementary Fig. S1c). Cooling rates ranging from − 3 to − 10 °C/min employed with either CS5 or CS10 yielded similar morphology and viability results (Supplementary Fig. S1c,d). Since a faster cooling rate may be more favorable for industrial production logistics, − 10 °C/min and CS10 were selected as the cryopreservation parameters for subsequent process optimization.

Cryopreserved cells are hypertonic relative to the aqueous rinse solutions that are typically employed to remove cryoprotectants post-thaw, which can lead to osmotic stress and cell-swelling, the primary causes of cell death during the thawing process^24^. To investigate a suitable rinse medium, four solutions approved for clinical use were examined: Normal Saline, Lactated Ringers, Balanced Salt Solution (BSS), and BSS PLUS. Normal Saline and Lactated Ringers are isotonic solutions typically used for intravenous administration, whereas BSS and BSS PLUS are irrigation solutions designed for ocular applications. REPS rinsed with BSS or BSS PLUS maintained characteristic RPE cuboidal morphology with high viability (95%) 1 DPT, while REPS rinsed in Normal Saline or Lactated Ringers had significantly reduced viability (*P* < 0.05) or poor morphology 1 DPT, respectively (Supplementary Fig. S2a,b). Healthy RPE cells secrete the neurotrophic factor pigment epithelium-derived factor (PEDF) in vivo, and PEDF secretion increases in vitro as RPE cultures polarize and mature^11,26,27^. No significant differences were detected among the rinse conditions for levels of secreted PEDF by 7 DPT (*P* = 0.41), and REPS from all conditions developed pigmented, polygonal morphology by 21 DPT (Supplementary Fig. S2a,c).

In order to remove residual DMSO from cryopreserved REPS, a two-step rinse procedure was developed. Immediately upon thaw, REPS were sequentially submerged into two fresh volumes of BSS. Analysis of the two rinse volumes after incubation with thawed REPS indicated a residual DMSO concentration of 0.014% (1.85 mM) in the first rinse solution, and < 20 µM DMSO (below the assay’s limit of detection) in the second rinse solution (Supplementary Fig. S2d). These results demonstrate significant removal of residual DMSO by the two-step rinse procedure.

In summary, REPS exhibit optimal post-thaw cell survival and expression of the RPE cell phenotype when cryopreserved at 7 DPS using a freezing rate of − 10 °C/min and a commercially available, xeno-free cryoprotectant (CS10) in conjunction with post-thaw rinsing in an irrigating solution approved for clinical use (BSS) (Fig. 1f).

### Cryopreservation and thaw of clinical-grade REPS using the optimized protocol

**Figure 2:**
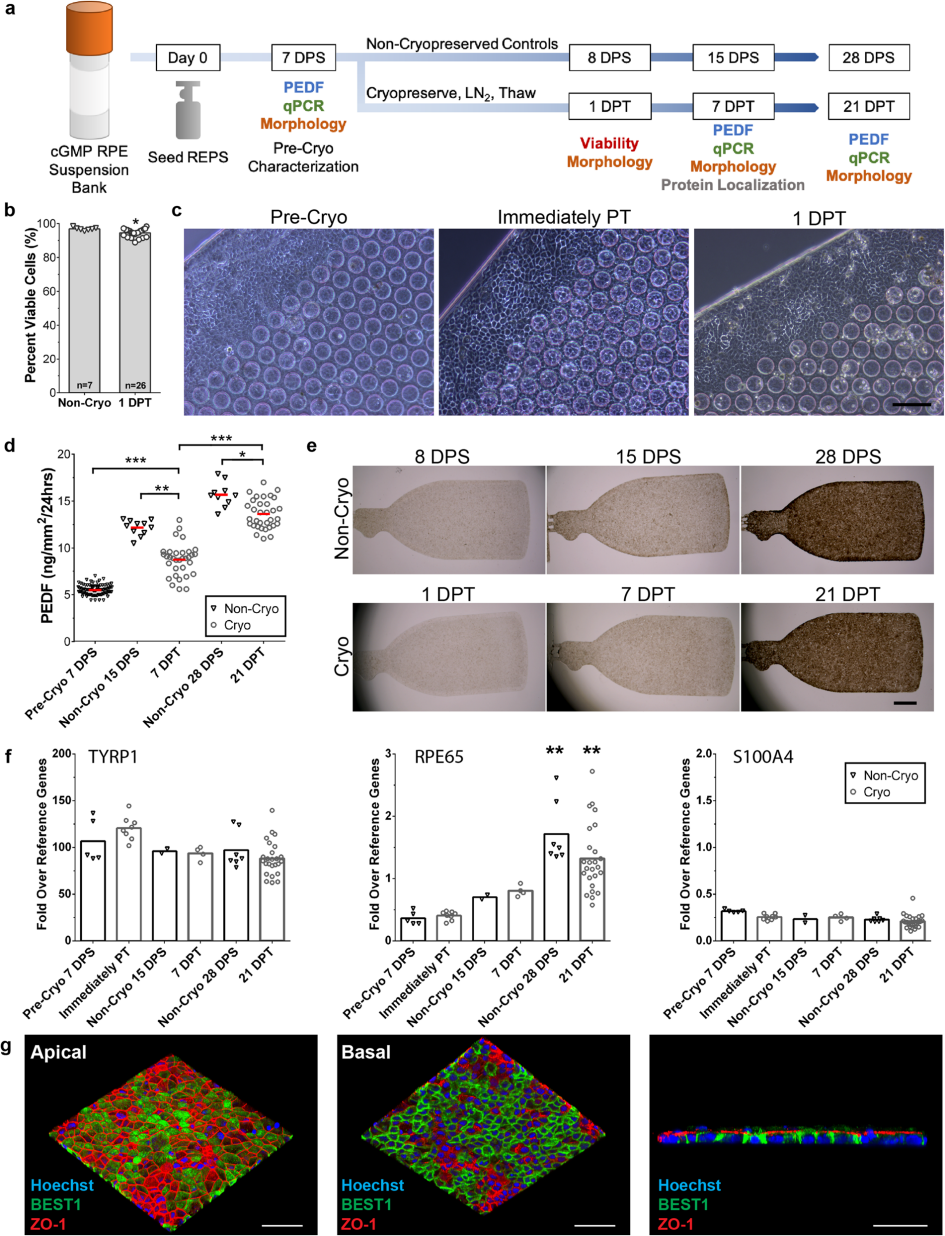
Cryopreserved REPS retain high viability, cellular identity, and secretory function post-thaw. (**a**) Schematic of overall research design indicates the characterization assays conducted on the designated day post-seeding (DPS) or day post-thaw (DPT). Non-cryopreserved REPS were maintained in culture to provide an age-matched control for each day of sample collection. (**b**) Cryopreserved REPS exhibit 95 ± 2.3% viability as measured by propidium iodide exclusion at 1 DPT (n = 26); viability of non-cryopreserved control REPS (n = 7) averaged to 97 ± 0.89% (mean ± SD., *P < 0.05, unpaired two-tailed t-test). (**c**) REPS exhibit epithelial, cuboidal morphology prior to cryopreservation (Pre-Cryo), immediately post-thaw (PT), and 1 DPT. (Phase contrast, scale bar, 100 µm.) (**d**) Amount of secreted PEDF by non-cryopreserved control REPS (black triangles) and cryopreserved/thawed REPS (gray circles) as measured by ELISA. Thawed REPS exhibited a significant increase in secreted PEDF at 7 DPT compared to Pre-Cryopreservation, and also at 21 DPT compared to 7 DPT (***P < 0.001). A significant difference was detected between thawed REPS and age-matched non-cryopreserved controls at 7 DPT (**P < 0.01) and at 21 DPT (*P < 0.05). (Pre-Cryo, n = 93; Non-Cryo, n = 10; Cryo, n = 33. Each data point represents the mean of two technical replicates. Horizontal red bar indicates mean of the biological replicates. Independent samples Kruskal–Wallis test with pairwise comparisons.) (**e**) Non-cryopreserved control REPS (Non-Cryo) and cryopreserved/thawed REPS (Cryo) display uniform pigmentation and similar coverage of the parylene scaffold over the course of the analysis (Bright field; scale bar, 1 mm). (**f**) RT-qPCR analysis of RPE marker genes (*TYRP1, RPE65*) and an EMT marker (*S100A4*) for non-cryopreserved control REPS (black triangles) and cryopreserved/thawed REPS (gray circles). No significant differences were observed between cryopreserved REPS and age-matched non-cryopreserved controls for each target gene (*P* > 0.05). A significant increase in *RPE65* was detected for both cryopreserved/thawed REPS and non-cryopreserved control REPS by 21 DPT or 28 DPS, respectively, compared to prior to cryopreservation and immediately post-thaw (**P < 0.01, independent samples Kruskal–Wallis test with pairwise comparisons). (**g**) En face (left, middle) and orthogonal (right) renderings of immunostained apical (ZO-1) and basolateral (BEST1) markers in cryopreserved REPS after 7–10 DPT. (Confocal Z-stacks, scale bar, 50 µm.) Error bars indicate standard deviation.

We next sought to determine whether clinical-grade cells could be similarly cryopreserved using the optimized parameters. Accordingly, REPS were seeded using a bank of RPE cells that was produced using current good manufacturing practices (cGMP) and released for clinical application. Characterization was performed prior to cryopreservation at 7 DPS and at three time points post-thaw to monitor recovery and maturation as evidenced by viability, gene expression, cuboidal RPE cell morphology, and secretory function (Fig. 2a). Non-cryopreserved REPS were maintained in uninterrupted in vitro culture to provide age-matched controls for each sampling time point (Fig. 2a).

Post-thaw viability of REPS was assessed 1 DPT (Fig. 2b, Supplementary Fig. S3). There was no significant difference in cellular metabolism between cryopreserved REPS and the non-cryopreserved control at 1 DPT (*P* = 0.66) as measured by AlamarBlue reduction (Supplementary Fig. S3). Although a significant difference was observed between cryopreserved REPS and the non-cryopreserved control by propidium iodide exclusion (*P* < 0.05), the percentage of viable cells of post-thaw REPS (n = 26) remained high (95 ± 2.3%). Non-cryopreserved control REPS (n = 7) exhibited an average viability of 97 ± 0.89%. Cryopreservation did not disrupt the cuboidal morphology of the RPE cells, and the monolayer of thawed REPS was similar in appearance to that present prior to cryopreservation (Fig. 2c).

To monitor the secretory function of cryopreserved clinical-grade REPS, the concentration of the neurotrophic factor PEDF in REPS-conditioned medium was quantified by ELISA prior to cryopreservation at 7 DPS (n = 93) and at two time points post-thaw: 7 DPT and 21 DPT (n = 33). The amount of PEDF secreted by REPS significantly increased (*P* < 0.001) from pre-cryopreservation levels (5.5 ± 0.53 ng/mm^2^/24 h, mean ± SD) within the first week post-thaw, as measured at 7 DPT (8.7 ± 1.7 ng/mm^2^/24 h) (Fig. 2d). By 21 DPT, cryopreserved/thawed REPS also demonstrated a significant increase (*P* < 0.001) in the amount of secreted PEDF (14 ± 1.6 ng/mm^2^/24 h) when compared to 7 DPT (Fig. 2d). Age-matched non-cryopreserved control REPS were assessed at 15 DPS and 28 DPS (n = 10) and secreted comparatively higher levels of PEDF (12 ± 0.82 and 16 ± 1.3 ng/mm^2^/24 h, respectively) compared to the thawed REPS (Fig. 2d). Thawed REPS secreted significantly less PEDF at 7 DPT (*P* < 0.01) and 21 DPT (*P* < 0.05) when compared to the age-matched non-cryopreserved control, but a trend of increasing PEDF secretion over time by thawed REPS was observed (72.5% and 87.5% of controls, respectively). Furthermore, by visual inspection the cryopreserved/thawed REPS acquired pigmentation similar to that of control REPS over the course of the analysis (Fig. 2e). These data suggest that cryopreserved REPS retain secretory function and mature post-thaw despite the cryogenic disruption of culture.

Further evidence of the capacity of REPS to mature post-thaw was demonstrated by a significant increase (*P* < 0.01) in the expression of the mature RPE marker gene *RPE65* by 21 DPT compared to the expression level prior to cryopreservation and immediately post-thaw (Fig. 2f). There were no significant differences between thawed REPS and the age-matched non-cryopreserved control for the expression of genes abundant in early (*TYRP1*) and mature (*RPE65*) RPE cells for any of the time points tested (Fig. 2f). Thawed REPS also expressed similarly low levels of the EMT marker *S100A4* at all sampling time points (Fig. 2f).

To evaluate the impact of cryopreservation on epithelial monolayer polarity, REPS were immunostained 7–10 DPT for the tight junction marker, ZO-1, and the Bestrophin-1 anion channel, BEST1, which respectively display apical and basolateral distribution in polarized RPE cells^28,29^. Thawed cells exhibited polarized localization of both markers (Fig. 2g), and quantification of nucleus Z-position indicated 92.1% basally localized nuclei consistent with a high degree of epithelial polarization (Supplementary Fig. S4)^30,31^. Taken together these results demonstrate that cryopreserved REPS exhibit normal gene expression, secretory function, epithelial polarity, cellular pigmentation, and characteristic RPE polygonal morphology post-thaw.

#### Stability of cryopreserved REPS throughout 1 year of storage in LN_2_

**Figure 3:**
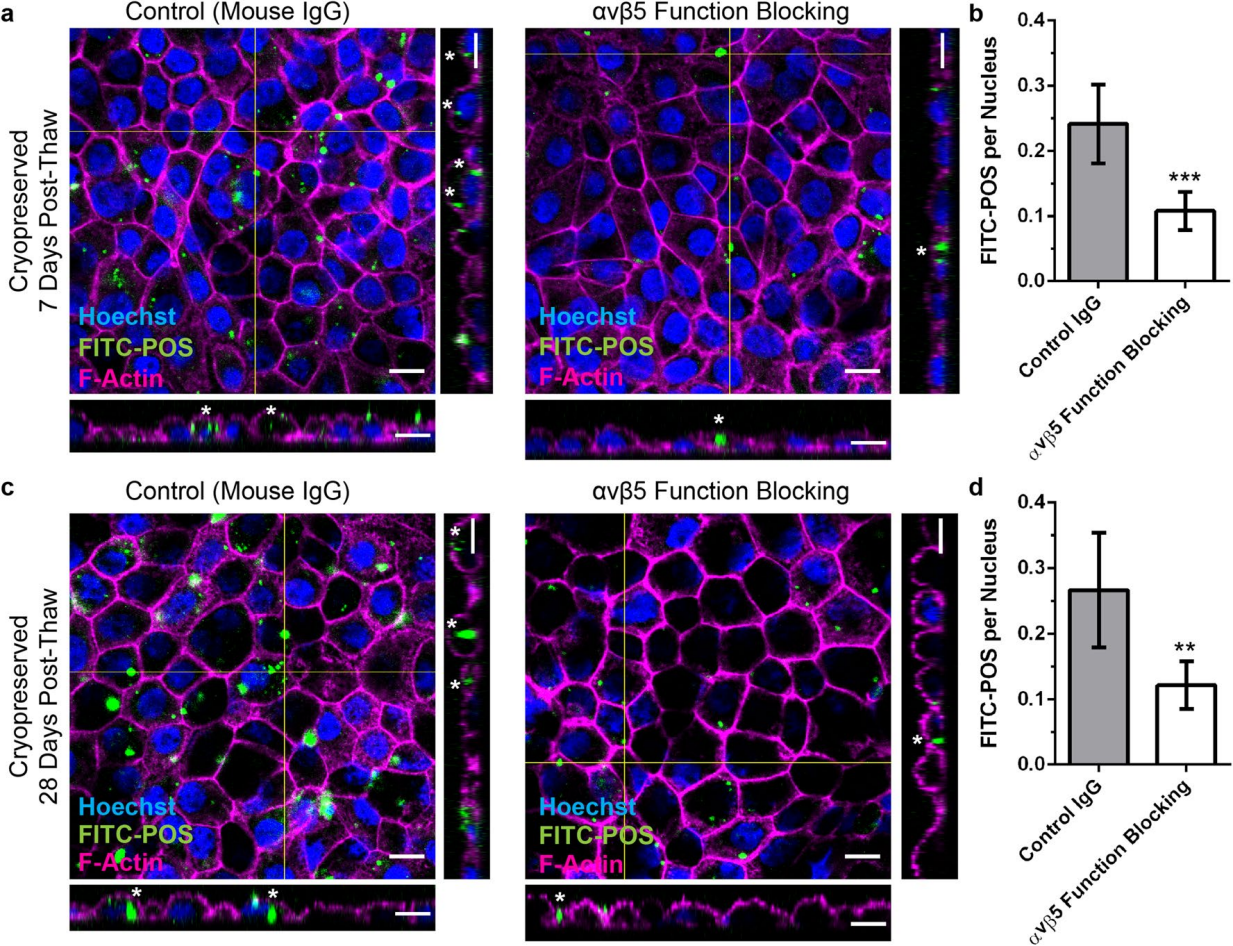
Cryopreserved REPS exhibit phagocytic activity in vitro after 15 months of LN_2_ storage. (**a**) Confocal fluorescence images and orthogonal projections of cryopreserved REPS cultured for 7 DPT and challenged for 16 h with FITC-labeled bovine POS in the presence of either control mouse IgG1 (left) or mouse ɑvβ5-integrin function blocking antibody (right). Asterisks in orthogonal projections indicate cells containing internalized FITC-POS. (**b**) Quantification of FITC-POS foci per nucleus for 7 DPT REPS. Six representative 20X confocal z-stacks were analyzed for each treatment condition. (**c**) Confocal images and orthogonal projections of cryopreserved REPS cultured for 28 DPT prior to the in vitro phagocytosis assay. (**d**) Quantification of FITC-POS foci per nucleus for 28 DPT REPS. All scale bars, 10 µm. Statistical significance was assessed using a two-tailed unpaired t-test (**P < 0.01, ***P < 0.001).

One of the fundamental advantages of a cryopreserved product is an extended shelf life. Therefore, the stability of cryopreserved REPS maintained in long-term LN_2_ storage was assessed for periods of 1 week, 6 months, and 1 year (n = 3 per time point). There were no significant differences observed among the storage periods for post-thaw viability, morphology, and gene expression. REPS retained > 92% viability 1 DPT and exhibited similar morphology compared to non-cryopreserved control REPS (Supplementary Fig. S5a,c). By 21 DPT REPS from each of the LN_2_-storage periods acquired characteristic cellular pigmentation and were comparable to non-cryopreserved control REPS for the expression of RPE marker genes (*TYRP1*, *RPE65*) as well as low expression of the EMT marker *S100A4* (Supplementary Fig. S5b,d). To further characterize the RPE phenotype of REPS cryopreserved for 15 months, in vitro phagocytosis assays were conducted at 7 DPT and 28 DPT using FITC-labeled bovine photoreceptor outer segments (FITC-POS). After incubation of 7 DPT REPS with FITC-POS, internalized FITC-POS were readily apparent in orthogonal projections of the REPS monolayer (Fig. 3a). Furthermore, the number of FITC-POS present was significantly reduced by co-incubation with a function blocking antibody against ɑvβ5-integrin (*P* < 0.001), which is an integrin receptor required for efficient phagocytosis of POS by RPE cells (Fig. 3b)^32–34^. Similar results were observed for REPS cultured for 28 DPT prior to the phagocytosis assay (Fig. 3c,d). Together these data provide proof-of-concept for the stability of REPS stored in LN_2_ through one year as demonstrated by high viability and retention of characteristic RPE phenotype and function post-thaw.

#### Cryopreserved REPS survive, mature, and demonstrate phagocytic function in vivo

**Figure 4:**
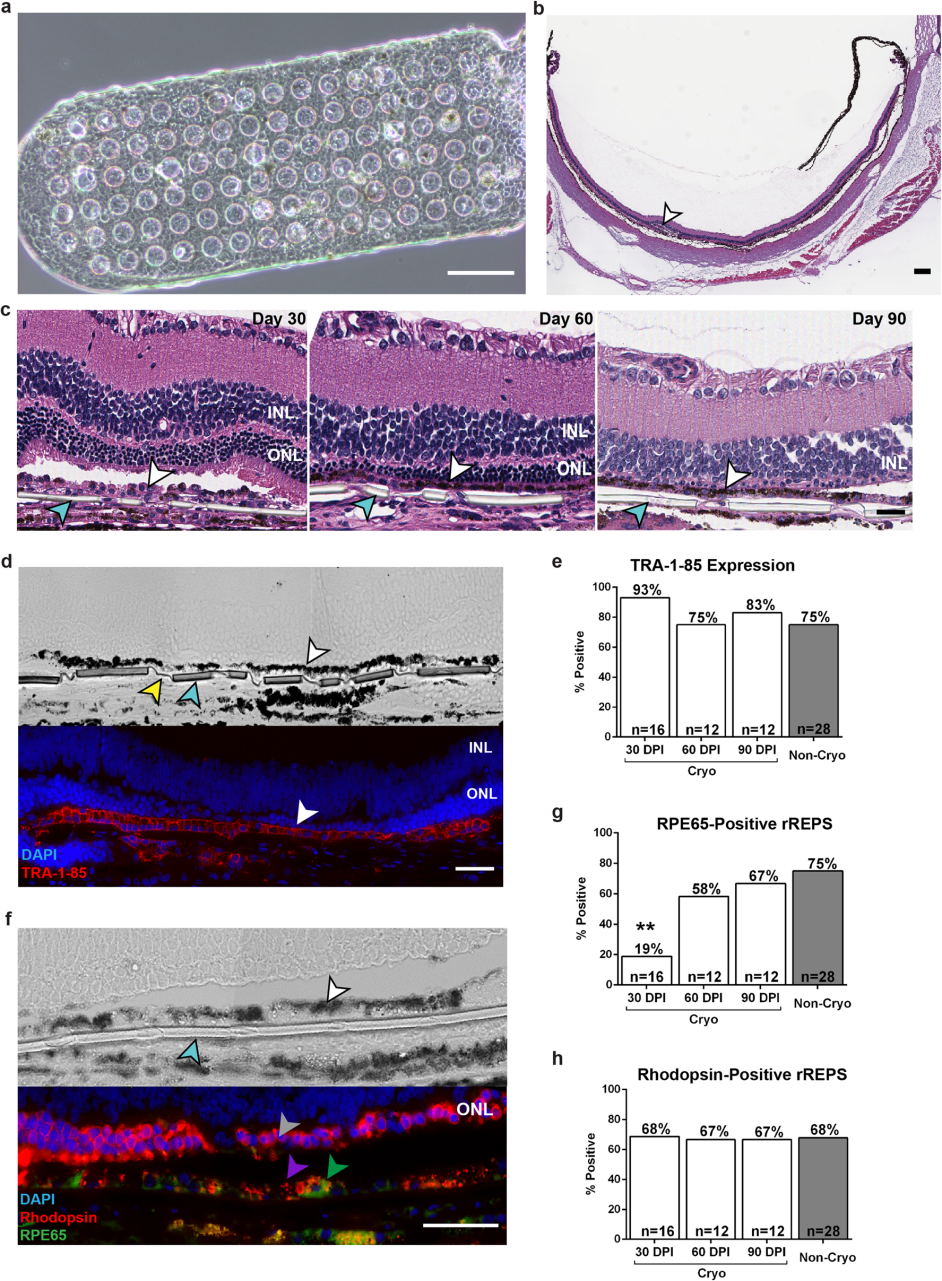
The rREPS implanted immediately post-thaw survive, mature, and demonstrate phagocytic function in vivo. (**a**) Reduced length/width dimensions of the parylene scaffold to accommodate implantation of rREPS into the rat subretinal space (phase contrast; scale bar, 100 µm). (**b**) Representative image of the posterior eye cup demonstrating subretinal location of implanted rREPS (arrow) (H&E stain; scale bar 100 µm). (**c**) Representative sections of eyes implanted with cryopreserved/thawed rREPS. Assessment at 30, 60, and 90 days post-implantation (DPI) shows an intact RPE monolayer (white arrows) associated with the parylene scaffold (teal arrows) (H&E stain; scale bar, 50 µm). (**d**) Adjacent histological sections representative of thawed rREPS at 60 DPI. Top panel: Bright field image showing RPE cells as a pigmented monolayer (white arrow). Submicron regions (yellow arrow) and mesh-supported regions (teal arrow) of the parylene scaffold are indicated^17,18^. Bottom panel: The RPE monolayer exhibits immunolabelling for the pan-human antigen TRA-1-85 (white arrow). Scale bar 50 µm. (**e**) rREPS immunolabelled for the pan-human marker TRA-1-85 are present in the majority of implanted rats for all cryopreserved/thawed time points and the non-cryopreserved control. (**f**) Adjacent histological sections representative of cryopreserved/thawed rREPS at 60 DPI. Top panel: Bright field image showing RPE cells as a pigmented monolayer (white arrow) supported by the parylene scaffold (teal arrow). Bottom panel: Phagocytic activity of rREPS as demonstrated by co-localization of rhodopsin-immunolabelled particulates (purple arrow) within implanted RPE65-immunopositive RPE cells (green arrow). Host photoreceptors also exhibit immunolabelling for rhodopsin (gray arrow). Scale bar, 50 µm. (**g**) Significantly fewer rats exhibit RPE65-immuonpositive rREPS at 30 DPI compared to control animals at 150 DPI. (**P < 0.01, 2 × 2 contingency table, Fisher’s exact test). (**h**) The percentage of rats that display co-localization of rhodopsin-positive particulates within RPE65-positive or TRA-1-85-positive RPE cells associated with the parylene scaffold is similar among cryopreserved/thawed time points and the non-cryopreserved control. Inner nuclear layer (INL), outer nuclear layer (ONL).

The ability to immediately administer cryopreserved REPS upon thaw would obviate the need for extended periods of culture post-thaw and would enable on-demand shipping to clinical sites. To demonstrate RPE cell survival, maturation, and function in vivo, cryopreserved REPS were thawed and immediately implanted into the subretinal space of the immunodeficient RCS/nude rat model of retinal degeneration^35^. The length and width of the parylene scaffold were reduced to accommodate the size of the rat subretinal space while the thickness and permeability of the scaffold remained unchanged^17^. The miniaturized rat REPS (rREPS) (Fig. 4a) were cryopreserved at 7 DPS using CS10 at − 10 °C/min, thawed, rinsed, and immediately implanted into the subretinal space (Fig. 4b). Implanted eyes were isolated at 30, 60, and 90 days post-implantation (DPI), and histological sections were analyzed for the expression of human and mature RPE markers by immunohistochemistry. Non-cryopreserved rREPS were examined at 150 DPI as a control. Cryopreserved/thawed RPE cells were observed as an intact monolayer supported by the parylene scaffold at each time point post-implantation (Fig. 4c). The human origin of the RPE cells was confirmed by TRA-1-85 immunolabelling of the pigmented monolayer associated with the scaffold (Fig. 4d)^36^. The percentage of rats exhibiting TRA-1-85-immunopositive rREPS was similar for each of the sampling time points of the cryopreserved/thawed condition as well as the non-cryopreserved control (Fig. 4e). The presence of a pigmented monolayer of human origin over the course of the analysis suggests that cryopreservation does not adversely affect the ability of RPE cells to remain adhered to the scaffold during surgery and implantation.

RPE65 is a critical enzyme that is necessary for the regeneration of the chromophore of the visual cycle, and RPE65 expression by RPE cells becomes more abundant during maturation and development^28,37^. The percentage of rats exhibiting RPE65-immunolabelled rREPS substantially increased over the post-implantation period (Fig. 4f,g). As observed 30 DPI, significantly fewer rats (*P* < 0.01) exhibited RPE65-immunopositive rREPS (19%). By 60 DPI and 90 DPI however, 58% and 67% of the implanted animals, respectively, demonstrated RPE65 immunoreactivity by the cryopreserved/thawed RPE cells associated with the parylene scaffold, which was comparable to the percentage of control animals (75%) assessed at 150 DPI that received non-cryopreserved rREPS (Fig. 4f,g). The substantial increase of animals that exhibited RPE65 immunolabelling over the course of the analysis suggests that RPE cells continue to mature in vivo after the cryopreserved rREPS are delivered into the subretinal space immediately post-thaw.

A primary function of mature RPE cells is the daily phagocytosis of shed photoreceptor outer segments (POS)^28^. Phagocytic function may be demonstrated in vivo by the presence of the photopigment rhodopsin within RPE cells as visualized by immunohistochemistry^38^. Phagocytosis by rREPS was determined by anti-rhodopsin immunolabelling of intracellular inclusions within RPE cells immunolabelled for either TRA-1-85 or RPE65 that were associated with the parylene scaffold (Fig. 4f, Supplementary Fig. S6). A comparable percentage of animals demonstrating phagocytic activity by rREPS was observed among each of the cryopreserved sampling time points and the non-cryopreserved control condition (Fig. 4h). Collectively, these data demonstrate that cryopreserved rREPS may be delivered immediately post-thaw into the subretinal space and that the RPE cells are able to survive as a monolayer, mature, and retain phagocytic function in vivo.

## Discussion

This report is the first to demonstrate successful cryopreservation of a substrate-adherent, hESC-derived RPE cell monolayer on an implantable scaffold. Cryopreserved REPS retain characteristic RPE cellular phenotype and secretory function in vitro as well as exhibiting high post-thaw viability after 1 year of storage in LN_2_. Furthermore, when rREPS are implanted into the subretinal space immediately post-thaw, the RPE cells demonstrate survival, maturation, and phagocytic function in vivo, which is crucial for a cellular therapy intended for administration upon thaw. We demonstrated that unpigmented REPS cryopreserved at 7 DPS have the capacity to mature post-thaw to form a polarized monolayer that exhibits mature RPE cellular functions and marker expression in vitro and in vivo.

We determined that the pivotal parameter for the effective cryopreservation of RPE cells is to perform freezing prior to the onset of pigmentation. REPS that manifested pigmentation prior to cryopreservation would undergo severe hyperpigmentation within the first 24 hours post-thaw, which was associated with poor RPE cell survival (Fig. 1c–e). This observation corroborates previous reports of attenuated growth and attachment^39^ or decreased efficacy^40^ of RPE cells in suspension that exhibit hyperpigmentation post-thaw as well as reports of other pigmented cell types with poor post-thaw recovery^41^. Our study is the first to associate post-thaw hyperpigmentation with decreased viability and reduced metabolism of RPE cells. This result is relevant to cellular therapies employing RPE cells^10–13^ as well as cultured epidermal autografts employed for the treatment of burn victims, which are reported to have depleted melanocyte populations due to cryopreservation^41^. A possible mechanism for post-thaw hyperpigmentation may be related to the response of RPE cells to reactive oxygen species (ROS). Both melanogenesis and stress from the cryopreservation/thaw processes have been reported to generate ROS, which can cause DNA damage^42,43^. Melanogenesis is also stimulated in response to DNA damage^42,44^, and the rapid hyperpigmentation observed post-thaw may be related to this phenomenon. Since the cumulative effects of necrotic and apoptotic pathways in response to freeze/thaw typically manifest within 6–24 hours post-thaw^25^, hyperpigmentation may occur while cells are still viable, and intracellular melanin persists within the RPE monolayer even after the RPE cells have died. Conversely, unpigmented RPE cells at 7 DPS have not manifested the melanogenic pathway and may not be subject to the potentially toxic effects of melanogenesis intermediates or their downstream sequelae. This may explain why suspensions of RPE cells are typically cryopreserved prior to pigmentation or while proliferative^45,46^. It is also possible that another factor expressed during early maturation of RPE cells concomitant with the onset of pigmentation is the primary cause of poor survival post-thaw, and melanogenesis is merely a response to that cytotoxic factor^42,44^. Indeed, neither 7 DPS nor 12 DPS REPS displayed pigmentation prior to cryopreservation, yet the more mature REPS exhibited significantly reduced expression of *RPE65* immediately post-thaw and poor morphology at 1 DPT (Supplementary Fig. S1a,b). Further investigation is needed to elucidate whether pigmentation or another facet of RPE cell maturation is responsible for the poor survival of melanized RPE cells in response to cryopreservation.

In order to demonstrate that the unpigmented REPS cryopreserved at 7 DPS are capable of maturation post-thaw, expression of mature RPE cell markers and evidence of secretory and phagocytic functions were assessed in vitro and in vivo. The significant increase (*P* < 0.001) of PEDF secretion over time as well as the apical localization of tight junction protein ZO-1 and the basolateral distribution of BEST1 protein and nuclei indicate that the adherent RPE cells matured to develop a polarized monolayer post-thaw (Fig. 2d,g; Supplementary Fig. S4)^11,26,27,29–31^. *RPE65* is a key marker of RPE cell maturation^28^, and in vitro expression significantly increased by 21 DPT compared to immediately post-thaw (Fig. 2f). Furthermore, the percentage of rats that exhibited RPE65-immunolabelled rREPS substantially increased after 30 DPI (Fig. 4f,g). Cryopreserved/thawed rREPS that were implanted into the subretinal space also demonstrated in vivo phagocytic function as evidenced by the co-localization of particulates immunolabelled for the photopigment rhodopsin within either RPE65-immunopositive cells or TRA-1-85-immunopositive cells associated with the parylene scaffold (Fig. 4f,h)^38^. In summary, cryopreserved/thawed REPS exhibited features of the “Four P” traits associated with mature RPE: pigmented appearance, polygonal morphology, polarized monolayer, and phagocytic activity^4^. We have demonstrated that REPS survive and mature in vivo in a rat model of retinal degeneration; survival and maturation of REPS administered to human patients with non-neovascular AMD will be assessed in future clinical trials. Taken together, these data demonstrate that unpigmented REPS cryopreserved at 7 DPS have the capacity to mature and perform characteristic RPE cellular functions in vitro and in vivo.

Development of a cryopreservation protocol for an adherent RPE-based therapeutic has been identified as a crucial milestone for Phase II and Phase III clinical trials as well as for commercialization of the implant^4^. Direct comparison to non-cryopreserved REPS suggests that cryopreserved/thawed REPS are comparable, but not identical. Although the polygonal cell morphology was not affected by cryopreservation, we observed slight but statistically significant reduction (*P* < 0.05) in viability 1 DPT for cryopreserved/thawed REPS compared to the age-matched control (95% vs. 97%, respectively) (Fig. 2b). We also observed significantly lower levels of PEDF secretion at 7 DPT (*P* < 0.01) and 21 DPT (*P* < 0.05) (Fig. 2d). However, the increasing trend for PEDF secretion over time (72.5% and 87.5% of non-cryopreserved controls at 7 DPT and 21 DPT, respectively) suggests that cryopreserved REPS may recover to be comparable to non-cryopreserved implants (Fig. 2d). Indeed, there was no significant difference in animals exhibiting RPE65-immunopositive REPS between the non-cryopreserved condition measured at 150 DPI and either of the cryopreserved/thawed conditions measured at 60 DPI (*P* = 0.77) or 90 DPI (*P* = 0.96) (Fig. 4f,g). Although not identical to the control, cryopreserved/thawed REPS exhibit RPE cell identity and function as well as providing logistical advantages for production, distribution, and commercialization.

As advancements are made in the field of regenerative medicine, the need to cryopreserve implants comprised of other therapeutic cell types is likely to escalate. Previous investigations have reported that rapid cooling rates (e.g. − 10 °C/min) during cryopreservation adversely affect stem cell aggregates^47^ and some mammalian monolayers^20,48^ possibly due to intracellular ice formation^24^ precipitated by uneven distribution of cryoprotectants and/or reduced capacity of cell aggregates/monolayers to extrude water. However, we observed no significant effect on the percentage of viable cells 1 DPT due to freezing rate (Supplementary Fig. S1d). This may suggest that the range of compatible cooling rates for a particular implant may depend on the dynamics of fluid transport across the cellular membrane and the scaffold material^24^. However, we observed that the fastest cooling rate tested (− 10 °C/min) resulted in the qualitative improvement of cuboidal RPE cell morphology 1 DPT (Supplementary Fig. S1c), which should be considered when determining a freezing rate that provides for immediate application of an adherent cellular therapy upon thaw.

Adequately removing cryopreservation medium from REPS prior to implantation is likely to be beneficial, and it has been reported that DMSO concentrations of ≤ 0.1% (12.8 mM) do not adversely affect the function of the mammalian retina^49^. Here, we report that the DMSO concentration in the final rinse solution is < 20 µM (Supplementary Fig. S2d), which suggests that the cryopreservation medium was adequately removed from REPS and the risk of transferring residual DMSO into the eye is minimal. Employing either BSS or BSS PLUS as the post-thaw rinse solution yielded REPS with high viability (95%) and characteristic polygonal RPE cell morphology at 1 DPT, potentially due to the ocular specificity and/or the enhanced buffering capacities of these solutions (Supplementary Fig. S2a,b). Conversely, Normal Saline lacks a pH buffering component, and REPS rinsed in saline post-thaw exhibited significantly reduced survival 1 DPT (*P* < 0.05) (Supplementary Fig. S2b). Despite the differences observed 1 DPT among the rinse solutions, the levels of secreted PEDF by thawed/rinsed REPS at 7 DPT were not statistically different (*P* = 0.41), and all conditions developed pigmented, cuboidal morphology by 21 DPT (Supplementary Fig. S2a,c). This suggests that adherent RPE cells on implants may recover from moderate cell death post-thaw. However, cryopreservation/thaw procedures that yield REPS with superior viability and morphology 1 DPT are more appropriate when developing a therapy to be administered immediately upon thaw.

This report is the first to demonstrate successful cryopreservation of an adherent RPE cell monolayer on an implantable, biostable scaffold available for therapeutic application immediately post-thaw. This xeno-free method yields REPS that exhibit a high degree of viability as well as characteristic RPE cell phenotype and function post-thaw as demonstrated in vitro and in vivo. Cryopreservation of an adherent RPE cellular therapy extends the product’s shelf life and allows for broad clinical distribution, enabling the treatment of wide-spread degenerative eye diseases such as AMD.

## Methods

### REPS

REPS were generated by seeding custom-designed parylene support matricies^17,18^ with frozen suspension stocks of RPE cells that were generated by spontaneous differentiation^45^ from WA09 human embryonic stem cells (WiCell Research Institute). The GMP-compliant RPE suspension banks were generated at the Center for Biomedicine and Genetics at City of Hope, Duarte, California, USA. Parylene scaffolds^17,18^ were coated with vitronectin sourced from human plasma (Corning) at a concentration of 3–5 μg/cm^2^. RPE cells were seeded at 1.5–2.9E5 cells/cm^2^ to achieve optimal post-thaw morphology and viability. In vitro culture employed X-VIVO 10 medium (Lonza) at 37 °C, 5% CO_2_.

### Cryopreservation of REPS

REPS were transferred from culture to 0.5 mL (Sigma) or 1.2 mL (Corning) cryovials containing CryoStor (BioLife Solutions) formulations with 2%, 5% or 10% DMSO (CS2, CS5, and CS10, respectively). REPS-loaded cryovials were stored on ice for up to 20 min, then transferred into a pre-chilled CryoMed controlled rate freezer (Thermo Fisher Scientific) set to 0 °C. Freezing was performed at constant rates of − 1 °C/min, − 3 °C/min, − 5 °C/min, or − 10 °C/min to a final temperature of − 90 °C. The chamber was held at − 90 °C until cryovials were transferred to a liquid nitrogen cryogenic storage vessel.

### Thaw of REPS

Frozen cryovials were maintained in liquid nitrogen for at least 1 day and up to 15 months. To thaw, cryovials were placed in a 37 °C water bath and REPS were removed from cryovials using fine-tipped forceps. Thawed REPS were rinsed in two sequential volumes (2 mL and 4 mL, respectively) of Normal Saline (Quality Biological), Lactated Ringers (Hospira), BSS (Alcon), or BSS PLUS (Alcon) at room temperature for approximately 20–30 s for the first rinse and approximately 1 min for the second rinse. Following the second rinse, REPS were transferred to X-VIVO 10 culture medium and maintained in a 37 °C incubator at 5% CO_2_.

### Viability assay

REPS were stained using 1:250 Hoechst 33342 (Life Technologies) and 1:1000 propidium iodide (PI) (Life Technologies) diluted in DPBS or X-VIVO 10 for 15–25 min in a 37 °C incubator at 5% CO_2_. Stained REPS were transferred to a microscope slide and at least 3 representative fields of view were acquired under fluorescence illumination on an Olympus BX51 microscope. The total number of cells was determined for each field of view by quantification of Hoechst-stained cells using ImageJ FIJI (National Institutes of Health); the total number of quantified nuclei ranged between 24,000 and 40,000 nuclei per REPS. The number of PI-stained, non-viable cells was similarly determined and the non-viable percentage was calculated by division using the total cell number.

### AlamarBlue metabolic assay

AlamarBlue Cell Viability Reagent (Thermo Fisher Scientific) was used per manufacturer’s instructions. AlamarBlue solution was diluted 1:10 in X-VIVO 10 medium, and REPS were incubated for 4 h in a 37 °C incubator at 5% CO_2_ in triplicates. Relative fluorescence of supernatant samples was assayed in technical duplicates using a Synergy H1 Hybrid Multi-Mode Microplate Reader (BioTek) using Ex/Em wavelengths of 560/590 nm. Raw relative fluorescence (RF) output was blank-subtracted for each sample.

### Immunofluorescence

After thawing and in vitro culture for 7–10 days, REPS were fixed in 4% methanol-free formaldehyde for 30 min, permeabilized with 0.1% Triton X-100 (Roche) for 10 min, and blocked with 5% goat serum (Jackson ImmunoResearch) and 1% BSA (ThermoFisher Scientific) in PBS for 30 min. Primary antibody incubations were performed for 2 h at room temperature using 1:200 dilutions of rabbit-anti-ZO1 (40-2200, Thermo Fisher Scientific) and mouse-anti-Bestrophin 1 (MA116739, Thermo Fisher Scientific). Secondary antibodies AlexaFluor 594 AffiniPure goat anti-rabbit IgG (1:200; #111585144, Jackson ImmunoResearch) and AlexaFluor 488 AffiniPure goat anti-mouse IgG (1:200; #115545062, Jackson ImmunoResearch) were incubated for 1 h at room temperature, followed by staining with Hoechst 33342 (2 µg/mL) and where indicated AlexaFluor 647-labeled phalloidin (1:1,000; A22287, Thermo Fisher Scientific) for 15 min in PBS. REPS were mounted under #1.5 coverslips with ProLong Gold antifade and imaged on an Olympus FV1000 Spectral Confocal with a UPLSAPO 20X air objective (NA: 0.75) or a PLAPON-SC 60X oil objective (NA: 1.40). ImageJ FIJI (National Institutes of Health) and FluoRender (University of Utah Scientific Computing and Imaging Institute) were used for image visualization and analysis. Quantification of nucleus position was performed using 17 µm thick confocal Z-stacks acquired from two immunostained implants at 5 representative 60 × fields of view across each implant. The center of mass (z-position) for each cell nucleus was determined using the 3D Object Counter^50^ tool in ImageJ/FIJI (n = 1232 total nuclei). For quantification of phagocytosis, six representative 20 × fields per REPS (approximately 9000 to 11,000 nuclei per REPS) were processed to quantify both nuclei and FITC-labeled POS using the 3D Object Counter tool. Identical exposure and analysis settings were used for image capture and quantification across treatment conditions.

### PEDF ELISA

Samples of culture supernatant were generated by incubating each REPS in 0.5 mL of fresh X-VIVO 10 medium for 24 ± 1 h. Supernatant was collected, aliquoted, and stored at − 80 °C. Sandwich ELISAs for Pigment Epithelium-Derived Factor (PEDF) were performed using a commercially available kit according to the manufacturer’s instructions (XpressBio). Samples were thawed at room temperature and were diluted to fall within the linear range of the standard curve (1:500 for 7, 15 DPS and 7 DPT samples; 1:2,000 for 28 DPS and 21 DPT samples). Each sample was assayed in technical duplicates and optical densities of samples were read at 450 nm using a Synergy H1 Hybrid Multi-Mode Microplate Reader (BioTek). Concentrations of PEDF were calculated using a best fit linear regression standard curve plot (R^2^ > 0.95). A negative control of undiluted X-VIVO 10 medium produced lower background signals than the most dilute point on the standard curve (0.031 ng/mL).

### RT-qPCR

Lysates for RNA purification were produced by aspirating growth medium and triturating REPS in RLT Buffer (Qiagen). Lysates were stored at − 80 °C and homogenized upon thaw with QiaShredder columns (Qiagen). RNA was purified with the RNeasy kit (Qiagen) and included an on-column genomic DNA digestion step using RNase-free DNase (Qiagen). Reverse transcription was performed on 150 ng of total RNA using iScript Reverse Transcription Supermix (BioRad) and reactions were diluted twofold with PCR-grade water after cDNA synthesis. PCR reactions were prepared using TaqMan Gene Expression Master Mix (Thermo Fisher Scientific) according to the manufacturer’s instructions. The following TaqMan primer–probe sets were used (all Thermo Fisher Scientific): EIF2B2 (Hs00204540_m1), SERF2 (Hs00428481_m1), UBE2R2 (Hs00215107_m1), RPE65 (Hs01071462_m1), TYRP1 (Hs00167051_m1), S100A4 (Hs00243202_m1), PMEL17 (Hs01124465_m1), BEST1 (Hs00188249_m1) and RLBP1 (Hs00165632_m1). Reference gene normalization (∆Ct) for each RNA sample was calculated as: geometric mean Ct of reference genes (EIF2B2, SERF2, UBE2R2)—gene of interest Ct. Normalized linear expression values for each gene are represented as 2^∆Ct^.

### DMSO detection assay

A rapid colorimetric assay to measure residual DMSO concentration in REPS rinse solutions was optimized based on the previously described use of DMSO as a probe for quantification of hydroxyl radicals in biological samples^51^. A Fenton reaction is used to generate hydroxyl radicals, which oxidize DMSO in the sample to methanesulfinic acid (MSA). Subsequent reaction of MSA with a diazonium salt (Fast Blue BB) yields a bright yellow diazosulfone product, which can be extracted into organic solvents and quantified by spectrophotometry. After thawing REPS, rinse solutions were collected and stored at -80˚C prior to processing. Oxidation was performed by mixing undiluted rinse samples (and DMSO standards) with freshly-prepared 500 mM FeSO_4_ catalyst (Sigma-Aldrich) at a ratio of 4:1 followed by addition of 1:200 dilution of 980 mM H_2_O_2_ (Sigma-Aldrich, final concentrations: 100 mM FeSO_4_, 5 mM H_2_O_2_). The oxidation reaction was allowed to proceed for 10 min at room temperature followed by addition of 0.5 reaction volume of 30 mM Fast Blue BB dye (Sigma-Aldrich). The reaction of MSA with Fast Blue BB was allowed to proceed for 10 min at room temperature, and the diazosulfone product was extracted by adding 0.8 reaction volume of 3:1 toluene-butanol solution and vortexing for 60 s. After briefly allowing the mixture to separate, the organic phase was collected in a fresh tube, washed with 2 volumes of butanol-saturated H_2_O to remove unreacted Fast Blue BB, and centrifuged for 3 min at 500×*g*. The organic phase was transferred to a fresh tube and 0.35 volume of 19:1 pyridine-glacial acetic acid solution was added for color stabilization. Absorbance was measured at 420 nm, and DMSO concentrations were calculated from the standard curve using a best fit standard curve linear regression (R^2^ > 0.95). The lower limit of detection for this method was approximately 20 µM, and no substantial assay interference by other compounds in CryoStor 10 was observed.

### Bovine photoreceptor outer segment labeling

Isolated photoreceptor outer segments (POS) harvested from approximately 50 bovine eyes (InVision BioResources, Cat# 98740) were covalently labeled with fluorescein isothiocyanate (FITC) (Thermo Fisher Scientific), aliquoted, and stored at − 80 °C until further use as previously described^33,38^.

### REPS phagocytosis

Three days prior to the assay, thawed REPS were transferred to a fresh tissue culture plate and incubated in culture medium supplemented with 15% FBS (Atlas Biologics, EF-0500-A) at 37 °C and 5% CO_2_. At 7 DPT or 28 DPT, approximately three million FITC-labelled POS per REPS were added to the culture medium; treated REPS were incubated at 37 °C and 5% CO_2_ for 16 h. Thirty minutes prior to POS exposure and throughout the POS incubation, REPS were treated with either 60 µg/mL anti-ɑvβ5-integrin function blocking antibody (abcam, ab177004) or 60 µg/mL mouse IgG1 isotype control (Thermofisher, Cat# 14-4714-82). REPS were rinsed five times using PBS with Ca^2+^ and Mg^2+^ (Gibco) and fixed using 4% paraformaldehyde. Immunofluorescence and image quantification were performed by a blinded analyst as described above.

### Rat REPS

Double homozygous RCS/Nude rats characterized by RPE dysfunction due to the deletion in the Mer tyrosine kinase (MerTK) receptor that abolishes internalization of photoreceptor outer segments by RPE cells were generated as previously described^35^ and were implanted subretinally with dimensionally modified (0.4 mm × 0.9 mm) rat REPS^17^. All experiments were approved by the University of Southern California Institutional Animal Care and Use Committee (IACUC) and were performed in accordance with the National Institutes of Health Guide for the Care and Use of Laboratory Animals and the ARVO Statement for the Use of Animals in Ophthalmic and Vision Research. All procedures were conducted in compliance with the ARRIVE guidelines. Cryopreserved rREPS were stored in LN_2_ for 1–2 months, thawed, rinsed twice, and implanted through a transscleral incision into the subretinal space of anesthetized (ketamine/xylazine, IP) animals. Control non-cryopreserved rREPS were maintained in X-VIVO 10 medium in a 37 °C, 5% CO_2_ incubator until the time of implantation at 30–56 DPS. Implant placement was monitored using a surgical microscope and confirmed by optical coherence tomography (OCT), and only rREPS that exhibited subretinal or partial subretinal placement were considered for downstream analyses. Subretinal implantation surgeries were performed in rat pups on post-natal days 20–30.

### Euthanasia and eye isolation

Animals were euthanized in accordance with ARVO statement for the Use of Animals in Ophthalmic and Vision Research by direct intracardial injection of 0.2 cc Euthasol, at 30, 60, and 90 days post-implantation (DPI) for cryopreserved/thawed rREPS and 150 DPI for non-cryopreserved rREPS. Eyes were enucleated and fixed in Davidson’s fixative for 18–24 h, followed by a proximal limbus cut to remove anterior eye tissue. Posterior eye cups containing rREPS were processed by alcohol dehydration, embedded in paraffin and sectioned at 5 µm.

### Histology and immunohistochemistry

Deparaffinized sections were stained with Hematoxylin and Eosin (Medical Chemical Corporation, 310-787-6800). For IHC, tissue sections were deparaffinized at 60 °C for 30–45 min and rehydrated followed by heat-induced antigen retrieval for three minutes. Sections were blocked using 5% bovine serum albumin and 0.1% Triton X-100 diluted in PBS for 30 min at room temperature and immunostained overnight with primary antibodies against TRA-1-85 (1:100; MAB3195, R&D Systems), RPE65 (1:500; AB105366, Abcam) and Rhodopsin (1:400; AB3267, Abcam) at 4 °C. Sections were stained with secondary antibodies goat anti-mouse IgG conjugated with rhodamine (1:100; 115 025 146, Jackson Immunoresearch) and goat anti-rabbit IgG conjugated with FITC (1:00; 115 095 144, Jackson Immunoresearch) for one hour at room temperature. Sections were mounted with fluorescein-enhancing mounting medium with DAPI (Vector Laboratory) and imaged using an Ultraviewer ERS dual spinning disk confocal microscope (PerkinElmer) equipped with a C-Apochromat 10 × high dry lens, a C-Apochromat 40 × water immersion lens NA 1.2 and an electron multiplier cooled digital camera (CCD). Images were captured and processed using PerkinElmer Volocity imaging software.

### Statistical analysis

In vitro viability and gene expression data is presented as mean ± standard deviation. All statistical analyses were performed using GraphPad Prism 6 or IBM SPSS Statistics 20. Significance was denoted as *P < 0.05, **P < 0.01, ***P < 0.001 or ****P < 0.0001 unless otherwise noted, and *n* indicates the number of unique implants or animals analyzed. Comparison between two groups used t-tests (two-tailed, unpaired). Groups of three or more were compared using one-way ANOVA with Tukey’s correction for multiple comparisons when Levene’s test of equality of error variances was > 0.05. When equality of variances was rejected (Levene’s test < 0.05), groups of three or more were compared using independent samples Kruskal–Wallis test with pairwise comparisons. Frequency analysis of in vivo phenotypes was performed using Fisher’s exact test with a 2 × 2 contingency table and α = 0.05.

## Supplementary Information

Supplementary Legends.

Supplementary Figure S1.

Supplementary Figure S2.

Supplementary Figure S3.

Supplementary Figure S4.

Supplementary Figure S5.

Supplementary Figure S6.

## Data Availability

Datasets are available from the corresponding author on reasonable request; the datasets generated and/or analyzed during the current study are not publicly available since data is proprietary to RPT and subject to limited distribution requirements.

## Abbreviations

AMD: Age-related macular degeneration
BSS: Balanced salt solution
cGMP: Current good manufacturing practices
DMSO: Dimethyl sulfoxide
DPI: Days post-implantation
DPS: Days post-seeding
DPT: Days post-thaw
EMT: Epithelial-to-mesenchymal transition
hESC: Human embryonic stem cell
LN_2_: Liquid nitrogen
PEDF: Pigment epithelium derived factor
POS: Photoreceptor outer segments
PSC: Pluripotent stem cell
RCS: Royal College of Surgeons
REPS: Retinal pigmented epithelial cells on a parylene scaffold
rREPS: Rat REPS
RPE cells: Retinal pigmented epithelial cells
ROS: Reactive oxygen species
