# Supplementary material for: Xeno-free cryopreservation of adherent retinal pigmented epithelium yields viable and functional cells in vitro and in vivo
Source: Sci Rep. 2021 Mar 18;11:6286. doi: 10.1038/s41598-021-85631-6 (PMC7973769; doi:10.1038/s41598-021-85631-6)
Supplement: Supplementary file 1 — Supplementary Legends. [file 41598_2021_85631_MOESM1_ESM.docx]

**Xeno-free Cryopreservation of Adherent Retinal Pigmented Epithelium Yields Viable and Functional Cells *In Vitro* and *In Vivo***

Britney O. Pennington^1,2^*, Jeffrey K. Bailey^1,2^, Mohamed A. Faynus^1,2^, Cassidy Hinman ^1,2^, Mitchell N. Hee^3^, Rory Ritts^4^, Vignesh Nadar^2^, Danhong Zhu^5^, Debbie Mitra^5^, Juan Carlos Martinez-Camarillo^5^, Tai-Chi Lin^5^, Biju B. Thomas^5^, David R. Hinton^5^, Mark S. Humayun^5,6,7^, Jane Lebkowski^2^, Lincoln V. Johnson^2^, Dennis O. Clegg^1,2,4^

1. Center for Stem Cell Biology and Engineering, Neuroscience Research Institute, University of California, Santa Barbara, CA, USA
2. Regenerative Patch Technologies LLC, Portola Valley, CA, USA
3. College of Creative Studies, Biology, University of California, Santa Barbara, CA, USA
4. Department of Molecular Cellular and Developmental Biology, University of California, Santa Barbara, CA, USA
5. Department of Pathology and Ophthalmology, USC Roski Eye Institute, Keck School of Medicine of the University of Southern California, Los Angeles, CA, USA.
6. Department of Biomedical Engineering, Denney Research Center (DRB) of the University of Southern California, Los Angeles, CA, USA.
7. USC Dr. Allen and Charlotte Ginsburg Institute for Biomedical Therapeutics, University of Southern California, Los Angeles, CA, USA

***Corresponding author:**

Britney O. Pennington, PhD, 6131 Biology 2 Bldg 571; NRI, UC Santa Barbara, Santa Barbara, CA 93106; [bop@ucsb.edu](mailto:bop@ucsb.edu)

**Supplementary Figure S1 | Identification of optimal post-seeding culture period, freeze rate, and cryopreservation medium.** **a,** REPS were cryopreserved at 4, 7, and 12 days post-seeding (DPS-Cryo) in triplicate using a rate of -3°C/min in CryoStor 5 (CS5). Cuboidal morphology of RPE cells (white arrowheads) and fibroblastic morphology (asterisks) were observed prior to cryopreservation (Pre-Cryo) and 1 day post-thaw (DPT) (left and middle panels, phase contrast). Structural features of the parylene scaffold appear as a regular array of circles (black arrows)^19^. By 30 DPT, cellular pigmentation was observed among all conditions (right panels, bright field; scale bar, 100µm). **b,** Expression of RPE marker genes (*PMEL, RPE65* and *RLBP1*) and the EMT marker (*S100A4*) assessed prior to cryopreservation (Pre-Cryo), immediately post-thaw (PT), and at 30 DPT for REPS cryopreserved at 4, 7, or 12 DPS. Gene expression by non-cryopreserved control REPS was assessed at 30 days post-seeding (DPS). For REPS cryopreserved at 7 DPS, no significant differences (*P* >0.05) were detected for gene expression levels immediately post-thaw compared to 30 DPT. (**P* <0.05, ***P* <0.01 independent samples Kruskal-Wallis test with pairwise comparisons; ****P* <0.0001 univariate ANOVA with post-hoc Tukey multiple comparisons). **c,** REPS were cryopreserved at 7 DPS using CryoStor formulation (CS2, CS5, or CS10) at the specified cooling rates. Typical RPE polygonal morphology was observed 1 DPT for REPS cryopreserved using CS5 or CS10 and cooling rates of -3, -5, or -10°C/min, while CS2 medium yielded REPS that exhibited fibroblastic morphology 1 DPT (asterisks). (Phase contrast; scale bar, 100µm). **d**, No statistical differences were detected among the tested conditions for the percentage of non-viable cells when assessed at 1 DPT (*P*=0.078, independent samples Kruskal-Wallis test with pairwise comparisons). Error bars indicate standard deviation.

**Supplementary Figure S2 | Identification of optimal post-thaw rinse solution. a,** REPS were cryopreserved at 7 DPS in triplicate using CS10 at -10°C/min. REPS were rinsed in one of the four solutions post-thaw: Lactated Ringers, Normal Saline, Balanced Salt Solution (BSS) or BSS PLUS. By one day post-thaw (DPT), REPS exhibited either fibroblastic cell morphology (white asterisks), clusters of non-viable cells (red asterisk) or typical RPE cuboidal morphology (white arrows). Cells from all conditions exhibited typical RPE polygonal morphology by 7 DPT and acquired pigmentation by 21 DPT (top two rows, phase contrast; bottom row, bright field; scale bar, 100µm). **b,** REPS rinsed with saline yielded significantly more non-viable cells 1 DPT compared to the other solutions (**P*<0.05, independent samples Kruskal-Wallis test with pairwise comparisons). **c,** No statistical differences were detected among the rinse conditions for secretion of PEDF by 7 DPT (*P*=0.41, univariate ANOVA with post-hoc Tukey multiple comparisons). Horizontal bar indicates mean. **d,** Triplicate REPS were rinsed immediately post-thaw in two sequential volumes of fresh BSS. The concentration of residual DMSO was significantly lower in the second rinse volume and was below the assay’s limit of detection (<20µM). (**** *P* <0.0001, unpaired two-tailed T-test). Error bars indicate standard deviation.

**Supplementary Figure S3 | Non-Pigmented REPS maintain cellular metabolism post-thaw.** REPS were cryopreserved at 7 days post-seeding (DPS) and thawed using the optimized conditions. Cellular metabolism was assessed at 1 day post-thaw (DPT) using AlamarBlue Viability Reagent and compared to age-matched, non-cryopreserved control REPS. No significant difference in cellular metabolism was observed between cryopreserved / thawed REPS and the control. (*P*=0.66, unpaired two-tailed T-Test). Relative Fluorescence Units (RFU).

**Supplementary Figure S4 | Cryopreserved REPS retain apical/basal cell polarity post-thaw**. Cryopreserved REPS were thawed and examined for apical/basal polarity of the RPE monolayer after 7-10 days of *in vitro* culture. Confocal Z-stacks (average depth: 17.5µm) of immunostained REPS were acquired at ≥5 representative 60X fields of view across each implant. Analysis of the centroid position of each cell nucleus indicated that 92.1% of nuclei (±5.4%, N=1,232 total nuclei quantified) were located in the basal half of Z-stacks, indicating a high degree of monolayer polarization. Dotted vertical line indicates midpoint of Z-stacks.

**Supplementary Figure S5 | Stability of REPS after one-year of storage in liquid nitrogen (LN_2_).** REPS were cryopreserved in triplicate 7 days post-seeding (DPS) using CS10 medium at -10°C/min. **a,** One day post-thaw (DPT), REPS retain typical cuboidal RPE cell morphology and (**b**) acquire cellular pigmentation by 21 DPT for all cryogenic storage periods (Phase contrast (**a**); Bright field (**b**); Scale bar, 100µm). **c,** REPS stored in LN_2_ throughout one year retain high percentage (>92%) of viable cells 1 DPT. No statistical difference in post-thaw viability was detected among the storage periods and the non-cryopreserved control (*P*=0.09, univariate ANOVA with post-hoc Tukey multiple comparisons). **d,** Gene expression assessed at either 28 DPS or 21 DPT for non-cryopreserved or cryopreserved/thawed REPS, respectively. There was not a significant difference in gene expression between the non-cryopreserved control REPS and any of the cryopreserved/thawed REPS for the RPE markers *TYRP1* (*P*= 0.47) and *RPE65* (*P*=0.72) (Univariate ANOVA with post-hoc Tukey multiple comparisons). All conditions expressed similarly low levels of the EMT marker *S100A4* by 21 DPT (*P*= 0.68, independent samples Kruskal-Wallis test with pairwise comparisons). Error bars indicate standard deviation.

**Supplementary Figure S6 | Cryopreserved rREPS mature and function *in vivo*.**  Representative immunofluorescence and bright field images of cryopreserved / thawed rREPS that were implanted into the subretinal space of RCS rats and examined at 60 days post-implantation. **a-c, f-h,** Individual fluorescence channels of the merged image (**d, i**).  *In vivo* phagocytic activity by rREPS is demonstrated by rhodopsin-immunolabelled particulates (purple arrows) indicative of rhodopsin-containing phagosomes within RPE65-immunopositive RPE cells (green arrows) that are supported by the parylene scaffold (white asterisks). Host photoreceptors cell bodies (outer segments have degenerated) also exhibit immunolabelling for rhodopsin (gray arrows). (Panel (**d**) is also presented in Fig. 4f.) Scale bar, 50µm. **e, j**  Representative bright field images of an adjacent histological section relative to the sections presented in **a**-**d** and **f-i**, respectively. Implanted RPE cells appear as a pigmented monolayer (white arrows) supported by the parylene scaffold (black asterisk) in the subretinal space of the RCS rat. Scale bar, 50µm.
