# Supplementary figures and images for: Xeno-free cryopreservation of adherent retinal pigmented epithelium yields viable and functional cells in vitro and in vivo
Source: Sci Rep. 2021 Mar 18;11:6286. doi: 10.1038/s41598-021-85631-6 (PMC7973769; doi:10.1038/s41598-021-85631-6)

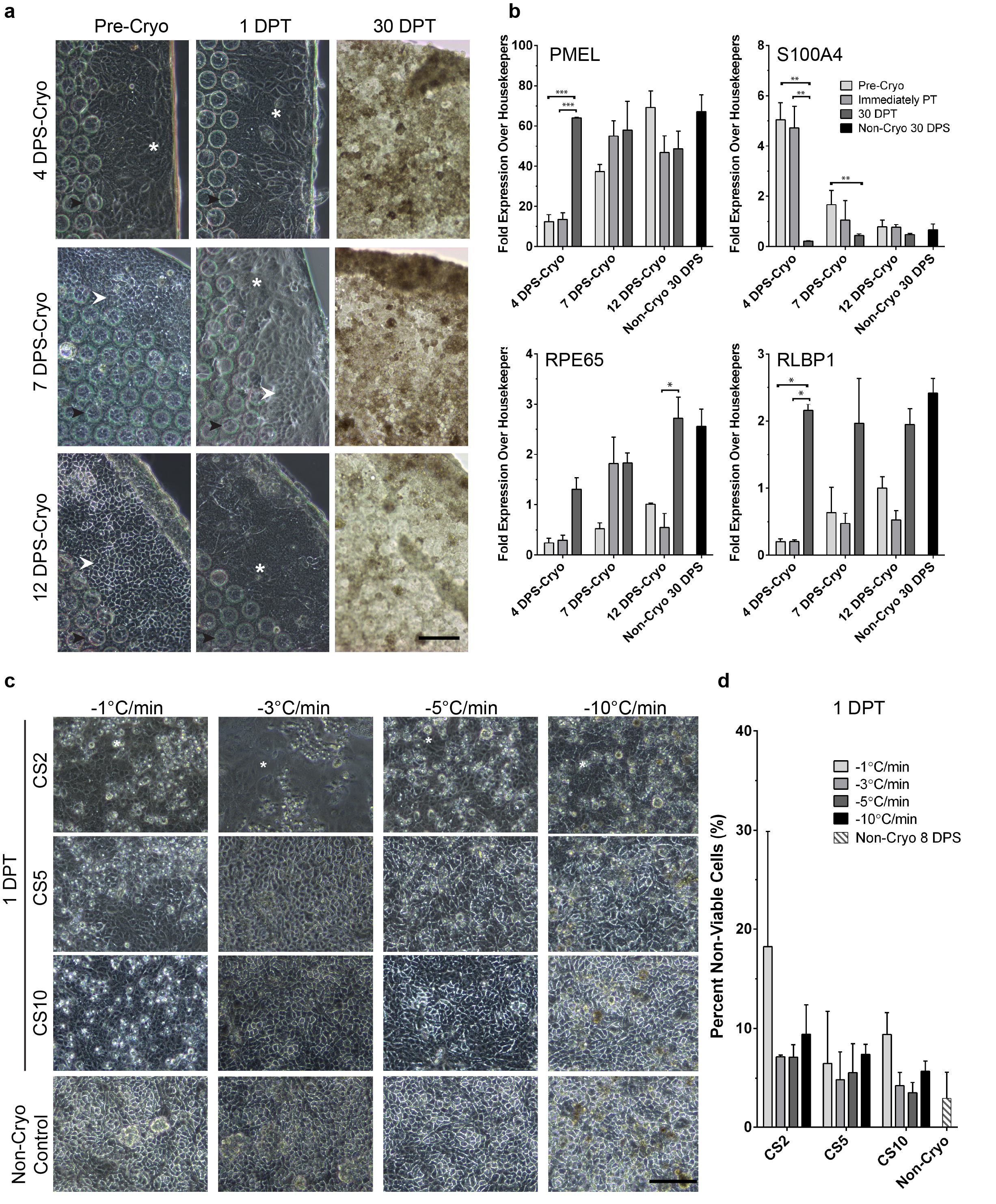

Supplement: Supplementary file 2 — Supplementary Figure S1. [file 41598_2021_85631_MOESM2_ESM.png]

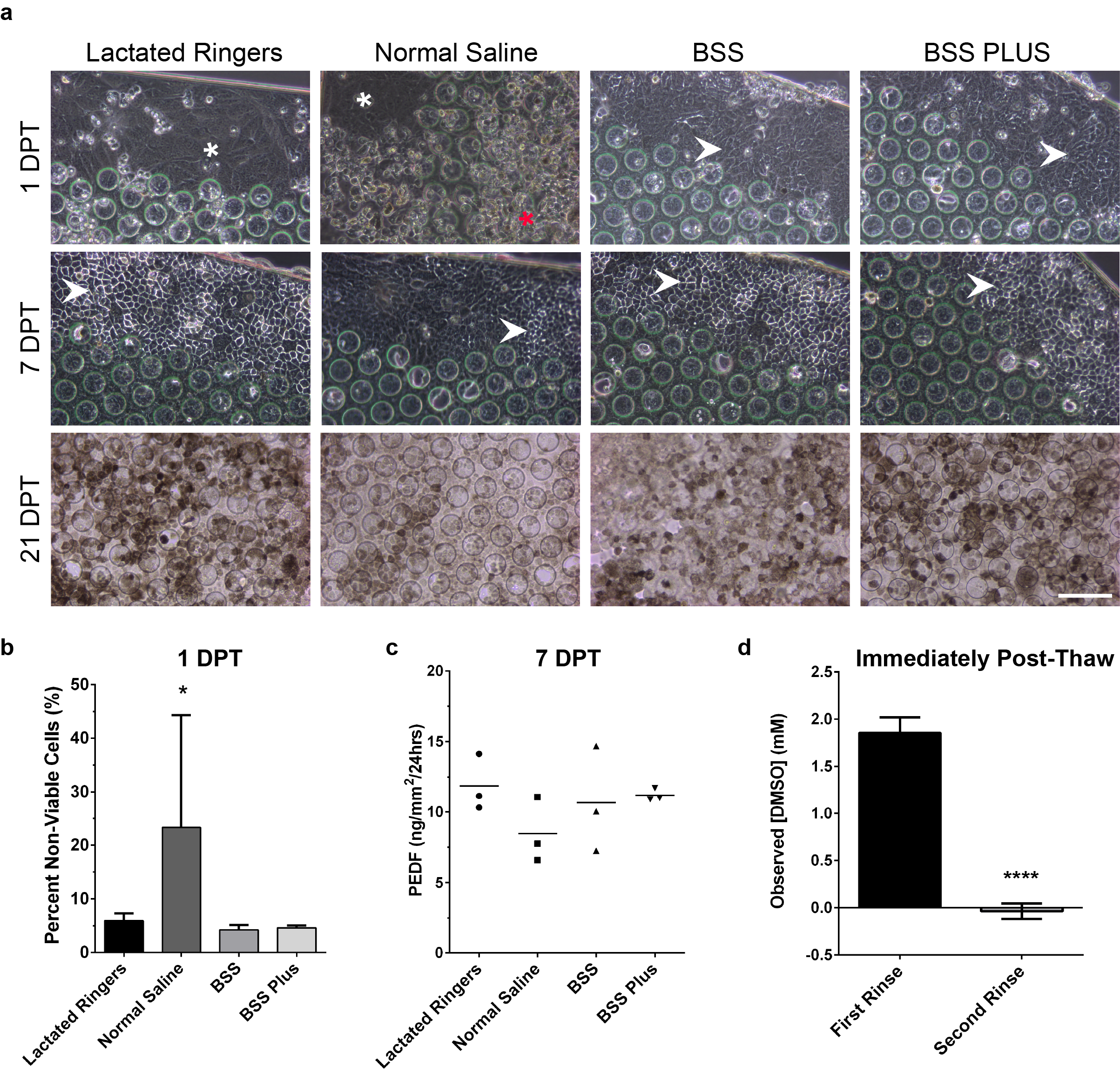

Supplement: Supplementary file 3 — Supplementary Figure S2. [file 41598_2021_85631_MOESM3_ESM.png]

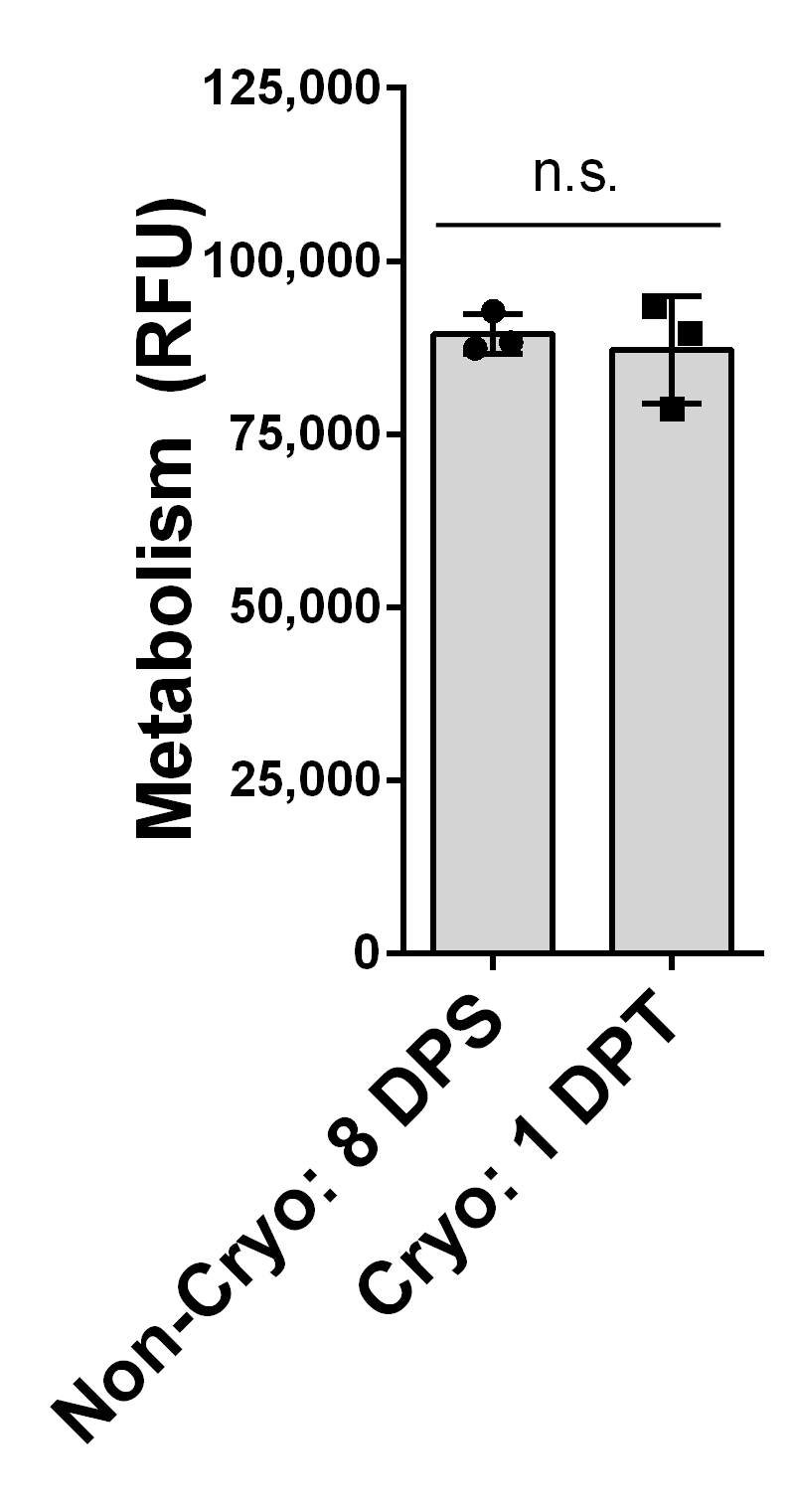

Supplement: Supplementary file 4 — Supplementary Figure S3. [file 41598_2021_85631_MOESM4_ESM.png]

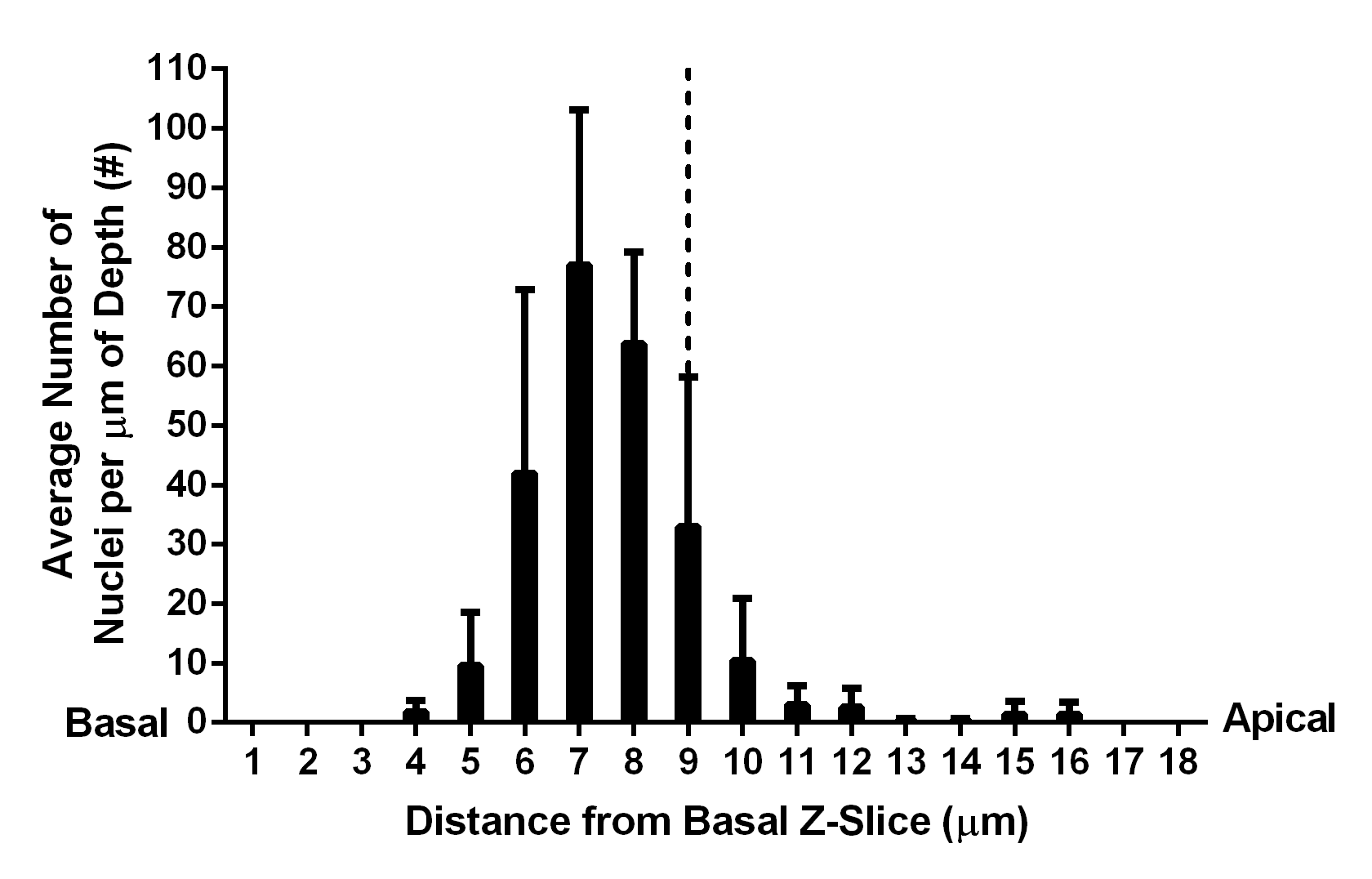

Supplement: Supplementary file 5 — Supplementary Figure S4. [file 41598_2021_85631_MOESM5_ESM.png]

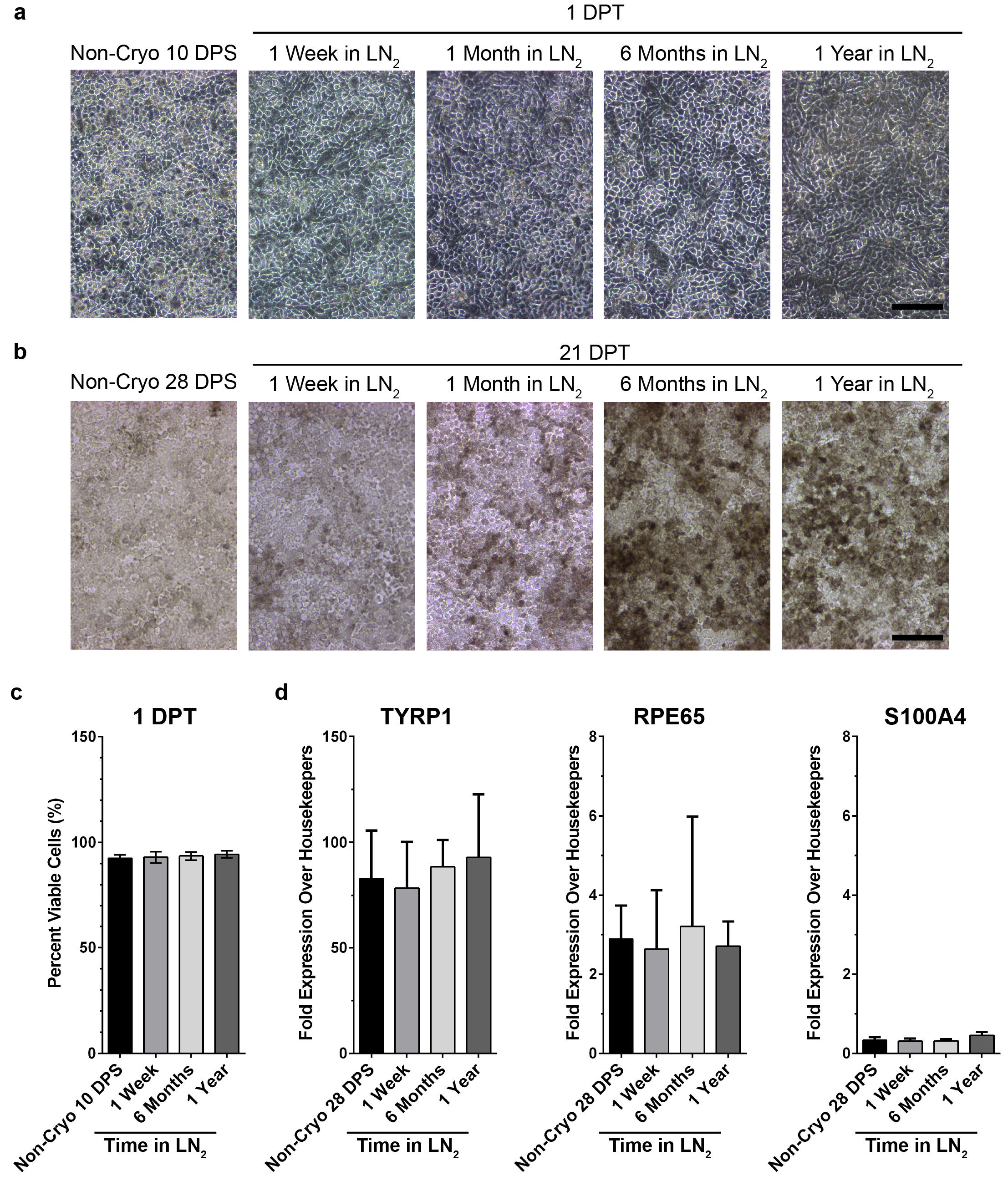

Supplement: Supplementary file 6 — Supplementary Figure S5. [file 41598_2021_85631_MOESM6_ESM.png]

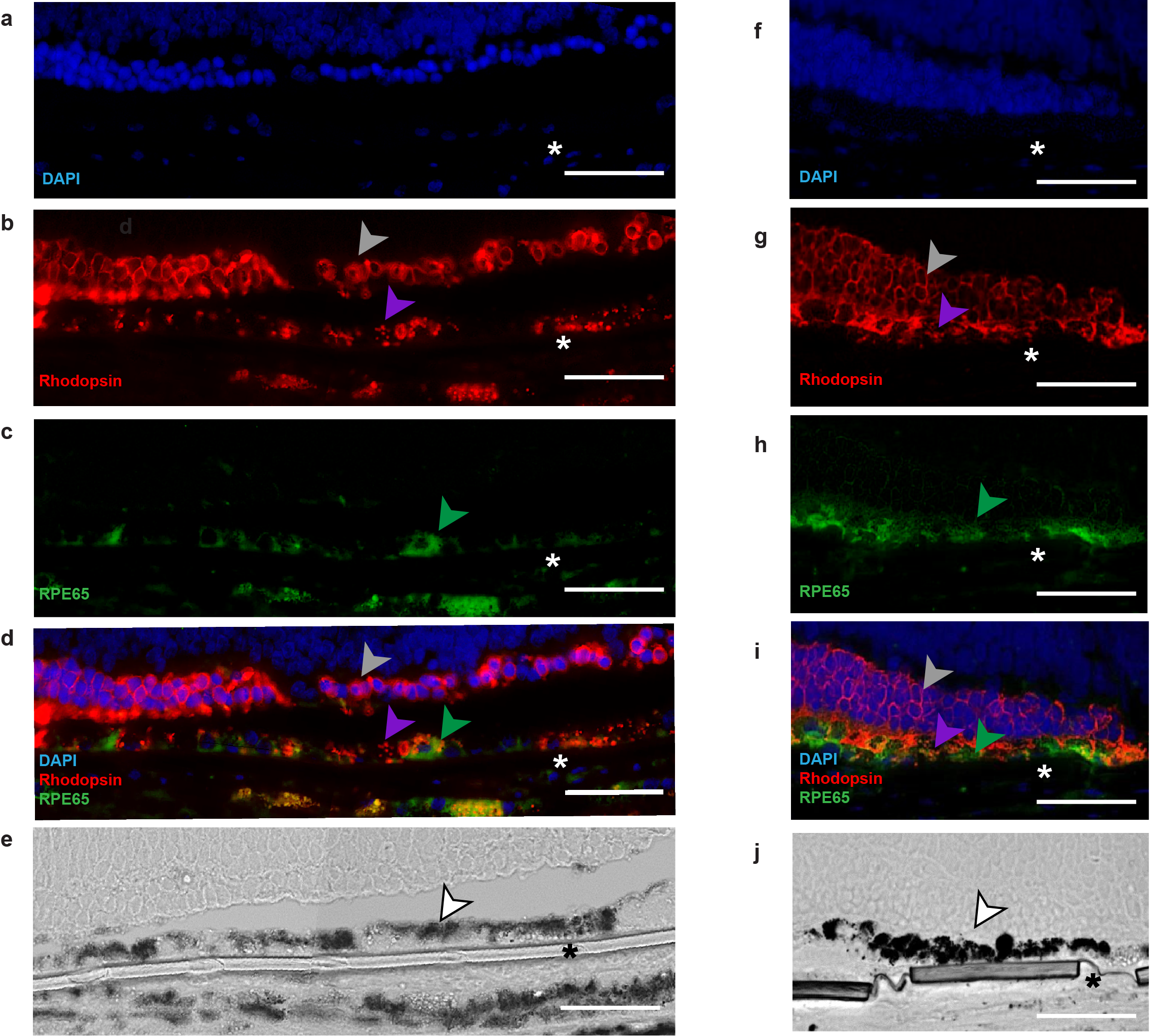

Supplement: Supplementary file 7 — Supplementary Figure S6. [file 41598_2021_85631_MOESM7_ESM.png]
